# Differentially expressed genes during the imbibition of dormant and after-ripened seeds – a reverse genetics approach

**DOI:** 10.1186/s12870-017-1098-z

**Published:** 2017-09-11

**Authors:** Farzaneh Yazdanpanah, Johannes Hanson, Henk W.M. Hilhorst, Leónie Bentsink

**Affiliations:** 1 Wageningen Seed Laboratory, Laboratory of Plant Physiology, Wageningen University, Droevendaalsesteeg 1, 6708 PB Wageningen, The Netherlands; 2 Umeå Plant Science Center, Department of Plant Physiology, Umeå University, SE-901 87 Umeå, Sweden; 3 Department of Molecular Plant Physiology, Utrecht University, Padualaan 8, 3584 CH Utrecht, The Netherlands

**Keywords:** *Arabidopsis thaliana*, *Delay of germination*, Knockout lines, Seed performance, Transcriptromics

## Abstract

**Background:**

Seed dormancy, defined as the incapability of a viable seed to germinate under favourable conditions, is an important trait in nature and agriculture. Despite extensive research on dormancy and germination, many questions about the molecular mechanisms controlling these traits remain unanswered, likely due to its genetic complexity and the large environmental effects which are characteristic of these quantitative traits. To boost research towards revealing mechanisms in the control of seed dormancy and germination we depend on the identification of genes controlling those traits.

**Methods:**

We used transcriptome analysis combined with a reverse genetics approach to identify genes that are prominent for dormancy maintenance and germination in imbibed seeds of *Arabidopsis thaliana*. Comparative transcriptomics analysis was employed on freshly harvested (dormant) and after-ripened (AR; non-dormant) 24-h imbibed seeds of four different *DELAY OF GERMINATION* near isogenic lines (*DOG*NILs) and the Landsberg *erecta* (L*er*) wild type with varying levels of primary dormancy. T-DNA knock-out lines of the identified genes were phenotypically investigated for their effect on dormancy and AR.

**Results:**

We identified conserved sets of 46 and 25 genes which displayed higher expression in seeds of all dormant and all after-ripened *DOG*NILs and L*er*, respectively. Knock-out mutants in these genes showed dormancy and germination related phenotypes.

**Conclusions:**

Most of the identified genes had not been implicated in seed dormancy or germination. This research will be useful to further decipher the molecular mechanisms by which these important ecological and commercial traits are regulated.

**Electronic supplementary material:**

The online version of this article (10.1186/s12870-017-1098-z) contains supplementary material, which is available to authorized users.

## Background

Freshly matured seeds usually exhibit primary dormancy, a trait defined as the failure of viable seeds to germinate under favourable conditions [8]. Seed dormancy plays a crucial role in the survival of plant species, but is also important for agricultural practice to prevent pre-harvest sprouting under cool, high humidity conditions [24]. Primary dormancy can be released by either cold stratification, which is a low-temperature treatment of imbibed seeds, or by an extended period of dry seed storage (after-ripening; AR) [9].

The transition from dormancy to germination is a critical step in the life cycle of plants [25]. The plant hormone abscisic acid (ABA) has long been known to play a major role in the establishment and maintenance of seed dormancy and the inhibition of seed germination, whereas gibberellins (GAs) and several other hormones, including brassinosteroids, ethylene, and cytokinins, have been shown to promote seed germination [38]. However, it is especially the balance between ABA and GA that controls the decision to germinate or not [19]. Mutations in genes regulating ABA levels or -sensitivity result in a reduced degree of seed dormancy [34, 35]. Whereas GA biosynthesis or sensing mutants result in a block of germination [23, 31, 33]. This hormonal control is also integrated with the seed’s responses to environmental conditions, such as light [45], temperature [55, 60] and nutrients [40].

Recent advances in gene expression analysis using microarrays allow genome-wide expression studies to characterize seed dormancy and germination [10, 20, 21, 26, 27, 36, 41, 52]. Carrera et al. [12] used a targeted transcriptomics approach in imbibed non-dormant mutants (*aba1* and *abi1*) compared to wild-type seeds that were or were not after-ripened. They concluded that, in Arabidopsis, after-ripening and dormancy are controlled by genetically separate pathways, and that ABA only affects the induction and maintenance of dormancy in imbibed seeds, but not after-ripening. The work also showed that application of exogenous ABA to after-ripened seeds does not mimic dormant seed states with respect to gene expression profiles. Recently it was shown that seed dormancy maintenance in the imbibed state was mainly controlled at the transcriptional level [3] and that transcriptional differences between dormant and non-dormant seed become visible already at early imbibition [17, 48].

Despite extensive research on dormancy and germination, many questions about the molecular mechanisms controlling these traits remain unanswered, likely due to its genetic complexity and the large environmental effects which are characteristic of these quantitative traits. Employing whole-genome scans for quantitative trait loci (QTL) is a common approach to identify genes involved in complex phenotypes. Particular attention in this method is given to the role of natural variation in the regulation of traits related to plant adaptation. Natural variation has been used to identify loci that control seed dormancy in nature. QTL analyses on six Recombinant Inbred Line (RIL) populations have identified eleven *DELAY OF GERMINATION* (*DOG*) QTL of which nine have been confirmed by near isogenic lines (NILs). The different *DOG* loci affect dormancy mainly by distinct genetic pathways as was concluded from the absence of strong epistatic interactions in the QTL analysis. This finding was confirmed by transcriptome analyses in freshly harvested dry seeds of the main *DOG*NILs, these lines showed distinct expression patterns compared to their genetic background Landsberg *erecta* (L*er*). The genes identified in the different *DOG*NILs represent largely different gene ontology profiles [6].

Here we aim at identifying genes that are required for dormancy maintenance and germination of imbibed seeds. Moreover, we focus on what is in common between the different pathways. For this the transcriptome of freshly harvested (dormant) and after-ripened (AR; non-dormant) 24-h imbibed seeds of the same set of *DOG*NILs and L*er* was investigated. We have identified sets of 46 and 25 genes that were up-regulated in seeds of all dormant (D-up) and all after-ripened (AR-up) *DOG*NILs and L*er*, respectively. We have investigated their role in seed performance by analysing knock-out (KO) mutants in these genes. With seed performance we refer to the capacity of seeds to germinate under various environmental conditions. Traits that contribute to seed performance are seed dormancy, seed longevity (as estimated in an accelerated aging test) and germination under stress conditions, such as high salt, osmotic stress and ABA treatment [32]. In this study we have characterised several genes affecting seed performance.

## Methods

### Plant material

The near isogenic lines of four *DELAY OF GERMINATION* (*DOG*) loci; *NILDOG1*-Cvi, *NILDOG2*-Cvi, *NILDOG3*-Cvi and *NILDOG6*-Kas-2 and Landsberg *erecta* (L*er*) were earlier described by Bentsink et al. [6]. Although for some of the *DOG* loci several NILs, containing introgression fragments from different accessions, were available we have chosen the ones with the strongest phenotypic effects. T-DNA insertional mutant lines and Columbia-0 (Col-0; N60000) were ordered from the European Arabidopsis Stock Center (NASC, www.arabidopsis.info). Details (SALK/SAIL entry, AGI code, knock out number and encoded protein) of T-DNA lines are provided in Additional file 1: Table S1.

### Growth conditions

NILs: Seeds were sown in Petri dishes on water-saturated filter paper, followed by a 4-day cold treatment at 4 °C, and transferred to an acclimated room at 25 °C with 16 h light/8 h dark for 2 days before planting in 7-cm pots with standard soil. Plants were grown in an air-conditioned greenhouse at 70% relative humidity, supplemented with additional light (model SON-T plus 400 W, Philips, Eindhoven, The Netherlands) providing a day length of 16 h light (long day), with light intensity 125 mmol m^−2^ s^−1^, and maintained at a temperature of 22–25 °C (day) and 18 °C (night). NILs were grown in a randomized complete block design with eight replicates. An experimental plot consisted of a row of 12 plants. At harvest the seeds of eight plants were bulked. Three of the eight replicates were used for the microarray analyses.

T-DNA knock-out lines: Lines were screened for homozygous insertions and grown with the wild type Columbia (Col) under greenhouse conditions using Rock wool supplemented with a Hyponex solution, in a randomized complete block design with four replicates per genotype.

### Sample preparation for microarray analyses

Dormant seeds were imbibed for 24 h in continuous light at 22 °C and then stored at −80 °C until RNA isolation. After-ripened seeds were imbibed for 24 h under the same conditions as the dormant seeds as soon as the seeds reached 100% germination in the germination experiment, also these seeds were stored at −80 °C until RNA isolation.

### Microarray analysis

Total RNA was prepared from 24-h imbibed seeds using RNAqueous columns with Plant RNA isolation aid (Ambion, Austin, TX, USA) according to the manufacturer’s protocol. The RNA was further purified through precipitations with isopropanol and a high salt solution containing 0.24 M sodium citrate and 0.16 M sodium chloride and subsequently with 2 M lithium chloride. RNA was qualitatively assessed and quantified using an Agilent 2100 Bioanalyzer with the RNA 6000 Nano Labchip® kit (Agilent, Santa Clara CA, USA) and Nanodrop1000™ spectrometry (NanoDrop Technologies, Inc., Wilmington, DE, USA). RNA was processed and cRNA synthesized according to the 3′ GeneChips OneCycle kit and hybridized on the ATH1 GeneChip (Affymetrix Inc., Santa Clara, CA, USA). The GeneChip data were analyzed using the R statistical programming environment and the Bioconductor packages [29, 51, 54]. The data was normalized using the RMA algorithm and a linear model was fitted to the data for comparisons of dormant to after-ripened seed within each genotype, the empirical Bayes method was used to reduce the gene wise sample variance [49]. The *P* values were then adjusted for multiple testing with the Benjamini and Hochberg method to control for false positives [5]. The microarray data were deposited in NCBI’s Gene Expression Omnibus (GEO number GSE90162). Microarray quality and reproducibility data is presented in Additional file 2: Figure S1. Dormancy up regulated genes (D-up) represent genes up-regulated (*P* > 0.0001) in the following comparisons, L*er* dormant vs L*er* after-ripened, NIL*DOG1* dormant vs NIL*DOG1* after-ripened, NIL*DOG2* dormant vs NIL*DOG2* after-ripened, NIL*DOG3* dormant vs NIL*DOG3* after-ripened, NIL*DOG6* dormant vs NIL*DOG6* after-ripened and vice versa After-ripening up regulated genes (AR-up) represent genes up-regulated (P > 0.0001) in the following comparisons, L*er* after-ripened vs L*er* dormant, NIL*DOG1* after-ripened vs NIL*DOG1* dormant, NIL*DOG2* after-ripened vs NIL*DOG2* dormant, NIL*DOG3* after-ripened vs NIL*DOG3* dormant, NIL*DOG6* after-ripened vs NIL*DOG6* dormant.

### T-DNA knock-out genotype analyses

A quick isolation method modified from [13] was performed to extract genomic DNA from leaves. In short, samples were ground in an extraction buffer containing 2 M NaCl, 200 mM Tris–HCl (pH 8), 70 mM EDTA and 20 mM Na2S2O5. The grinding was conducted with a stainless steel ball at 30 Hz for 1 min (96-well plate shaker, Mo Bio Laboratory). Then samples were incubated at 65 °C for 1 h. Supernatants were collected after centrifugation at maximum speed for 10 min. DNA was precipitated by adding iso-propanol and 10 M NH4Ac with ratio of 1:1/2:1 to the supernatant. This mixture was incubated at room temperature for at least 15 min, then centrifuged for 20 min at maximum speed. The DNA pellet was retrieved and rinsed with 70% ethanol followed by centrifugation for 5 min at maximum speed to recover the pellet. After drying, the DNA pellet was dissolved in distilled water. Homozygous T-DNA insertion lines were screened with gene specific primers (left and right) and insert border primers (Additional file 1: Table S1). T-DNA plants that amplified only the insertion product were consider to be homozygous mutants.

Polymerase chain reactions (PCR) were performed in a 12.5 μL-volume containing approximately 30 ng DNA, 25 μM of each dNTP, 25 ng of forward and reverse primers, 0.05 U of DNA polymerase (Firepol, Solis BioDyne), 312.5 μM of MgCl_2_. The reaction protocol was as follows; denaturation at 95 °C for 5 min followed by 30s at 95 °C, 30s annealing at 52 to 57 °C and a 45 s to 2 min extension at 72 °C, this cycle was repeated for 35 times, and ended with a final amplification for 10 min at 72 °C. The polymorphism was detected by agarose gel electrophoresis at concentrations from 1.5% and higher (*w*/*v*) depending on size of differences.

### Germination assays

Germination tests to follow the release of seed dormancy were performed as described by Alonso-Blanco et al. [2] with small adjustments. In short, at several time intervals during seed dry storage until all seed batches reached 100% germination aliquots of 50 to 100 seeds of each genotype were evenly sown on a filter paper soaked with 0.7 ml demineralized water in a 6-cm Petri dish. Petri dishes were placed in moisture chambers consisting of plastic trays containing a filter paper saturated with tap water and closed with transparent lids. Chambers were stored for 1 week in a climate chamber illuminated with 38-W Philips TL84 fluorescent tubes at 8 W m2 in continuous light at 22 °C. After that, the total number and the number of germinating seeds was scored and the percentage of germinating seeds was calculated.

Germination under stress conditions was performed on fully after-ripened seeds. Stress conditions were: osmotic stress (−1 MPa mannitol; Sigma-Aldrich), salt stress (130 mM NaCl; Sigma-Aldrich), ABA stress (0.15 μM ABA; Duchefa Biochemie). ABA was dissolved in 10 mM MES buffer (Sigma-Aldrich) and the pH adjusted to 5.8. To measure seed longevity, an accelerated aging test was performed by incubating seeds above a saturated ZnSO4 solution (40 °C, 85% relative humidity) in a closed tank for 5 days. Then the seeds were taken out and germinated on demineralized water as described before.

## Results

### Identification of seed dormancy and after-ripening up-regulated genes

**Fig. 1:**
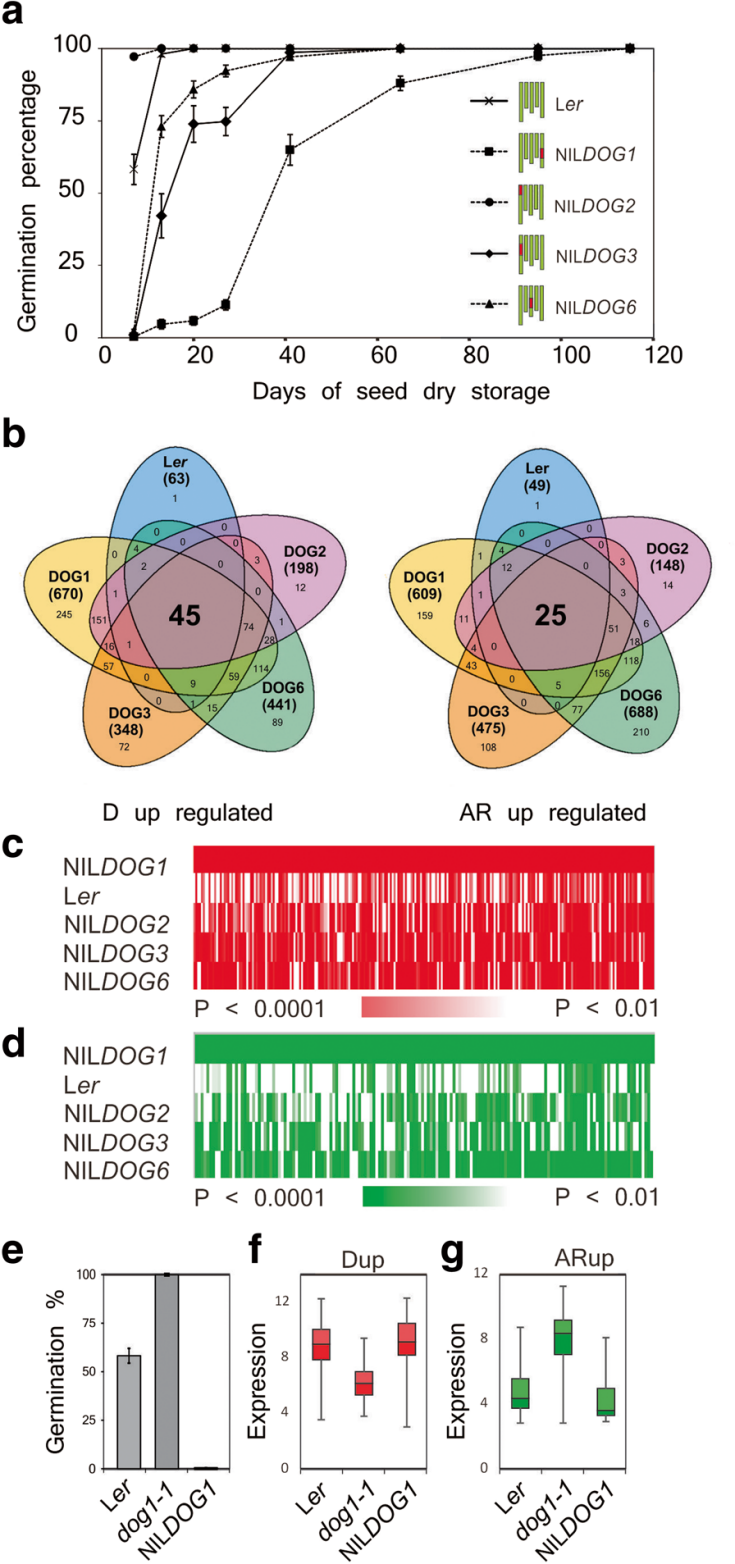
Microarray analysis of dormant and after-ripened seeds after 24 h of imbibition of five genotypes with differing dormancy levels; L*er*, NIL*DOG1*, NIL*DOG2*, NIL*DOG3* and NIL*DOG6*. **a** After-ripening requirement of the five genotypes. On the right graphical representations of the NILs are depicted showing the 5 chromosomes with the introgressed regions (in red) in an otherwise L*er* background (in green). **b** Venn diagrams showing the number of genes that are differentially expressed (*P* < 0.0001) in dormant (D-up) and after ripened (AR-up) 24-h imbibed seeds of different genotypes. For each genotype the total number of differential expressed genes is indicated between brackets. In the intersection of all genotypes the number of genes that are investigated in this study are presented, 46 and 25 for the D-up and AR-up set, respectively. **c** Heat map consisting of 245 NIL*DOG1* D-up genes (P < 0.0001). The significance of these genes in the other genotypes is indicated, the white color indicates the genes that are not significantly different in the other genotypes (*P* < 0.01). **d** Heat map consisting of 159 NIL*DOG1* AR-up genes (P < 0.0001). The significance of these genes in the other genotypes is indicated, the white color represents the genes that are not significantly different in the other genotypes (P < 0.01). **e** Germination behaviour of freshly harvested seeds of L*er*, *dog1* and NIL*DOG1*. **f** Box plot showing the expression of the 45 D-up genes in freshly harvested imbibed L*er*, *dog1* and NIL*DOG1* seeds (expression data taken from Dekkers et al. [16]). **g** Box plot showing the expression of the 25 D-up genes in freshly harvested imbibed L*er*, *dog1* and NIL*DOG1* seeds

Seeds of L*er*, NIL*DOG1*-Cvi, NIL*DOG2*-Cvi, NIL*DOG3*-Cvi and NIL*DOG6*-Kas-2 were investigated for their dormancy status. After-ripening was followed by performing germination tests during a time course of dry seed storage (Fig. 1a). After 120 days all genotypes had lost dormancy and showed 100% germination. NIL*DOG2* was less dormant and NIL*DOG3*, NIL*DOG6* and NIL*DOG1* were more dormant when compared to L*er*. Both dormant and after-ripened seeds of each genotype were sampled 24 h after sowing (HAS) for microarray analysis, allowing the comparison of the dormant and after-ripened seed transcriptomes of these five genotypes with varying levels of primary dormancy.

The transcriptome data was investigated to identify genes that are up-regulated in 24 h imbibed dormant (D) L*er*, NIL*DOG1*, NIL*DOG2*, NIL*DOG3* and NIL*DOG6* seeds and genes that are up-regulated in 24 h imbibed after-ripened (AR; non-dormant) seeds of the same lines. One thousand eight hundred ninety-six genes (*P* < 0.0001) were differentially expressed when performing within-genotype comparisons for the two stages analysed (dormant versus AR). In dormant seeds 63, 670, 198, 348, and 441 genes were up regulated and 49, 609, 148, 475 and 688 genes were up-regulated in AR seeds for L*er*, NIL*DOG1*, NIL*DOG2*, NIL*DOG3* and NIL*DOG6*, respectively (Fig. 1b).

A large proportion of the differentially expressed genes is specific for the genotypes analysed at *P* < 0.0001; however, these genes were differentially expressed in the other genotypes at lower significances. This has been visualised for the genes that are specific for NIL*DOG1* (Fig. 1 c,d). Most of the 245 NIL*DOG1* D-up and 159 NIL*DOG1* AR-up genes are differentially expressed (*P* < 0.01) in the other genotypes. This indicates that the genes that are specifically differentially expressed are based on quantitative expression differences rather than qualitative.

Genes that are important for dormancy and AR are expected to be differentially expressed between these stages in all genotypes tested (intersections in the Venn diagrams of Fig. 1b). This led to the identification of 45 up-regulated genes in all dormant genotypes (Dormancy-up; D-up; Table 1) and 25 genes that were up-regulated in all after-ripened genotypes (After-ripened–up; AR-up; Table 2). Further investigation of the expression patterns using the Seed EFP browser (http://www.bioinformatics.nl/efp/cgi-bin/efpWeb.cgi) revealed that, in general, all the genes that were up-regulated in dormant seeds at 24 HAI, were highly expressed in dry seeds, remained high during the imbibition of dormant seeds but were down regulated during the germination of AR seeds. Vice versa, genes that were up-regulated in AR seeds, had a low expression in dry seeds that increased with imbibition time (Additional file 2: Figure S2). Furthermore, the relation with dormancy becomes clear when the expression of the individual genes is investigated in imbibed seeds of L*er*, *dog1-1* and NIL*DOG1-*Cvi that have very clear dormancy differences (Fig. 1e). D-up genes are highly expressed in dormant L*er* and NIL*DOG1*-Cvi seeds, whereas AR-up genes are highly expressed in the non-dormant *dog1-1* mutant (Fig. 1 f,g).

**Table 1:** Mutants isolated from D-up genes

SALK/SAIL entry	AGI code	Knock out #	Encoded protein
SALK_073011C	AT2G29300	KO 1	NAD(P)-binding Rossmann-fold superfamily protein (RFSP?)
SALK_028749.55.25.x	AT2G31350	KO 2	Mitochondrial glyoxalase 2 (GLX2-5)
SALK_054451.53.45.x	AT2G33830	KO 3	Dormancy/auxin associated family protein(ATDRM2)
SALK_025507C	AT2G38800	KO 4	Plant calmodulin-binding protein-related (PCBP)
SALK_082639C	AT3G14880	KO 5	Transcription factor-related
SALK_150592C	AT5G01670	KO 6	NAD(P)-linked oxidoreductase superfamily protein
SALK_059351	AT5G64210	KO 7	Alternative oxidase2 (AOX2)
SALK_104275C	AT1G01240	KO 8	Unknown protein
SALK_110011C	AT1G05840	KO 9	Eukaryotic aspartyl protease family protein
SALK_027164C	AT1G27990	KO 10	Unknown protein
SALK_036898C	AT2G19900	KO 11	The malic enzyme1(ATNADP-ME1)
SALK_037108.56.00.x	AT1G13640	KO 12	Phosphatidylinositol 3- and 4-kinase family protein
SALK_101144	AT1G56600	KO 13	Galactinol synthase(GOLS2)
SALK_138905.29.65.x	AT2G27940	KO 14	RING/U-box superfamily protein
SALK_094895	AT3G02990	KO 15	Member of Heat Stress Transcription Factor family (HSFA1E)
SALK_025488.38.10	AT3G03310	KO 16	Lecithin:cholesterol acyltransferase 3 (LCAT3)
SALK_038352	AT3G22490	KO 17	Seed maturation protein
SALK_082777C	AT3G53410	KO 18	Paralog of ubiquitin E3 ligase (LUL2)
SALK_090239C	AT3G62090	KO 19	Phytochrome-Interacting Factors (PIF6)
SAIL_512_E03	AT4G19390	KO 20	Uncharacterised protein family
SALK_137617.43.90.x	AT5G02840	KO 21	LHY/CCA1-LIKE 1 (LCL1)
SALK_101433C	AT1G13340	KO 22	Regulator of Vps4 activity in the MVB pathway protein
SALK_025893C	AT1G20650	KO 23	Altered Seed Germination 5 (ASG5)
SALK_087702C	AT1G77450	KO 24	NAC domain-containing protein 32 (NAC032)
SALK_003223C	AT1G79440	KO 25	Succinate-semialdehyde dehydrogenase 1 (SSADH1)
SAIL_563_D10	AT1G80090	KO 26	Cystathionine beta-synthase family protein (CBSX4)
SALK_078702	AT3G50740	KO 27	UDP-glucosyl transferase 72E1 (UGT72E1)
SALK_116062C	AT3G53040	KO 28	Late embryogenesis abundant (LEA)protein
SALK_082087C	AT4G09600	KO 29	Gibberellin-regulated gene family(GASA3)
SALK_112631	AT4G20070	KO 30	Allantoate Amidohydrolase (AtAAH)
SALK_105045	AT4G25580	KO 31	CAP160 protein
SALK_043547C	AT4G36700	KO 32	RmlC-like cupins superfamily protein
SALK_135551C	AT5G65280	KO 33	GCR2-like 1 (GCL1)
SAIL_1256_F11	AT5G58650	KO 34	Plant peptide containing sulfated tyrosine 1(PSY1)

The table includes information about the affected genes (according to TAIR10^a^)

^a^TAIR database website: www.arabidopsis.org

**Table 2:** Mutants isolated from AR-up genes

SALK entry	AGI code	Knock out #	Encoded protein
SALK_043889	AT4G34135	KO 35	UDP-Glucosyltransferase 73B2 (UGT73B2)
SALK_070860C	AT3G26060	KO 36	PEROXIREDOXIN Q (PRXQ)
SALK_094069C	AT3G26570	KO 37	Phosphate transporter 2;1 (PHT2;1)
SALK_091600.51.00.x	AT5G49910	KO 38	Chloroplast heat shock protein 70–2 (CPHSC70-2)
SALK_097487C	AT4G34131	KO 39	UDP-glucosyl transferase 73B3 (UGT73B3)
SALK_086616C	AT3G20210	KO 40	Delta vacuolar processing enzyme (DELTA-VPE)
SAIL_547_D05	AT4G31330	KO 41	Protein of unknown function
SALK_007230.56.00.x	AT5G13400	KO 42	Peptide transporter 5
SALK_017818.55.50.x	AT2G45180	KO 43	Lipid-transfer protein/seed storage 2S albumin superfamily protein
SALK_095678	AT1G07890	KO 44	Ascorbate peroxidase 1 (APX1)
SALK_090550.52.85.x	AT1G47128	KO 45	Responsive to dehydration 21 (RD21)
SALK_015756	AT3G45010	KO 46	Serine carboxypeptidase-like 48 (scpl48)
SALK_132995.40.05.x	AT4G34260	KO 47	Altered Xyloglucan 8 (AXY8)

The table includes information about the affected gene (according to TAIR10^a^)^a^

TAIR database website: www.arabidopsis.org

Among the identified genes there were several that had been related to seed dormancy or germination before, including *PHYTOCHROME-INTERACTING FACTOR 6* (*PIF6, KO19, AT3G62090)*) [47], *GIBBERELLIN 3-OXIDASE 2* (*GA3OX2*) [59] and *ALTERED SEED GERMINATION 5* (*ASG5,* KO23, AT1G20650) [4]. In addition, we found genes encoding for late embryogenesis abundant (LEA) proteins which are known to accumulate during seed desiccation and in response to water deficit induced by drought, low temperature, or salinity [30, 43]. The identified genes cover various GO molecular function categories among which by far the largest proportion is enzyme-related, including transferase activity, kinase activity and hydrolase activity, next to nucleotide binding proteins, including transcription factors.

### Isolation of T-DNA mutants for genes involved in seed dormancy and germination

**Fig. 2:**
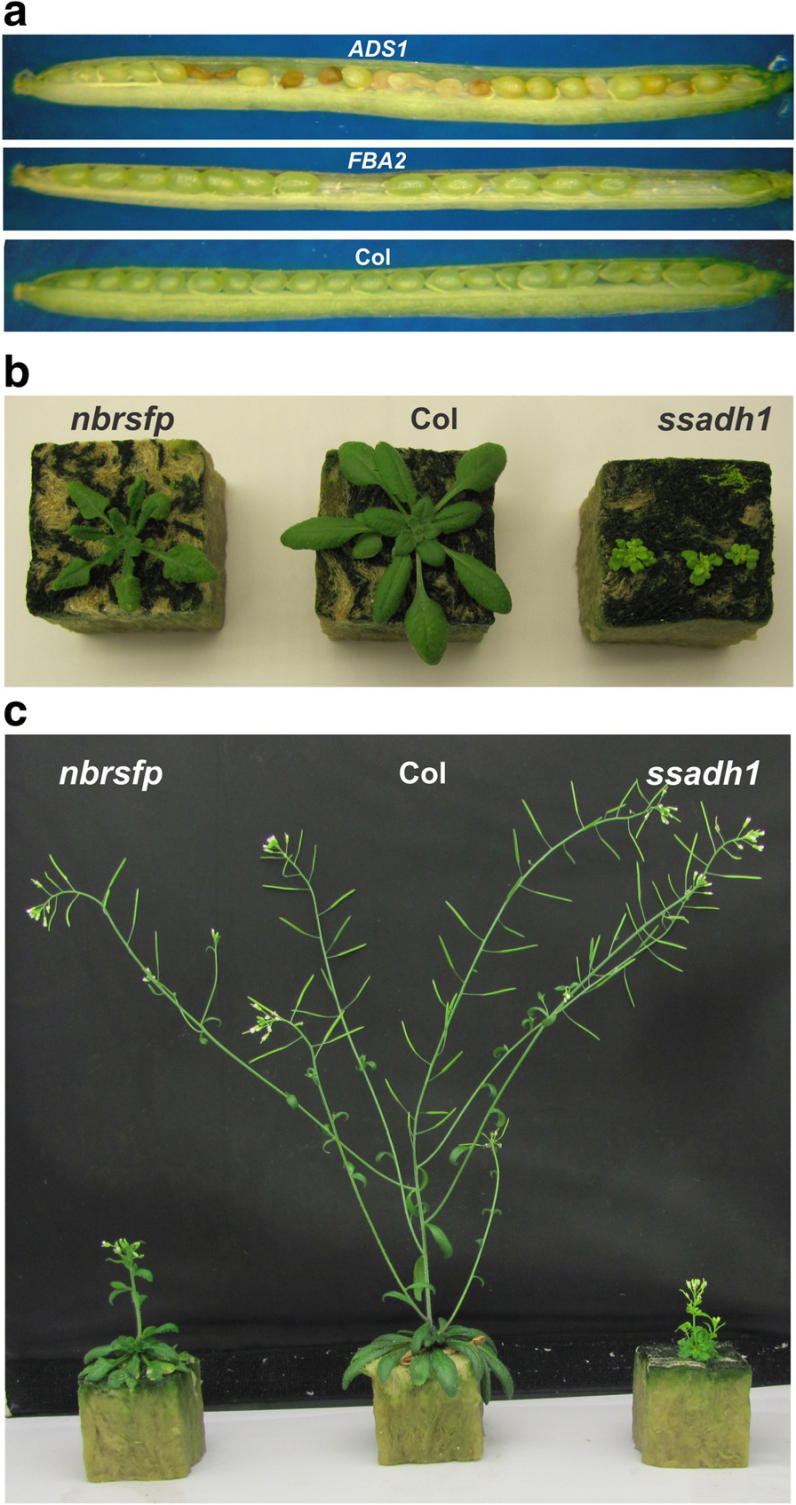
Plant phenotypes of T-DNA knock-out lines in comparison with wild type Columbia (Col). **a** Aborted seeds in siliques from heterozygous T-DNA lines with insertions in *FBA2* (AT1G06080) and *ADS1* (AT4G38970). **b** Four-week old plants of the NAD(P)-BINDING ROSSMANN-FOLD SUPER FAMILY PROTEIN (*nbrsfp*; KO1, AT2G29300) and *SUCCINATE-SEMIALDEHYDE DEHYDROGENASE* mutant (*ssadh1*, KO25, At1G79440) (**c**) *nbrsfp*, Col and *ssadh1* 6 weeks after germination

To investigate whether the identified genes indeed affect dormancy and AR we have analysed their T-DNA knock-out lines for seed performance phenotypes. For most of the identified genes, T-DNA mutants could be selected from the SALK and SAIL collections (NASC, http://arabidopsis.info/), but for eight genes no T-DNA insertion mutants were available (Additional file 1: Table S1). In all cases, homozygous lines were generated and confirmed using a PCR-based approach. For 47 genes a homozygous KO mutant could be selected. For nine genes (mostly in the AR-up set) no insertion was found in any of the plants genotyped (described as ‘all wild type’ in Additional file 1: Table S1). Moreover, for two genes *FRUCTOSE-BISPHOSPHATE ALDOLASE* (*FBA2*; AT4G38970) and *DELTA-9 DESATURASE1* (*ADS1* AT1G06080) T-DNA insertions were identified, but no homozygous mutants could be selected. Likely, the mutants homozygous for these genes are lethal; therefore siliques of these lines were dissected to investigate possible seed abortion. This confirmed that homozygous mutant seeds of these lines were aborted (around a quarter) at an early stage of seed development (Fig. 2). Complete genotyping information is given in Additional file 1: Table S1.

All the homozygous T-DNA lines were grown together with wild type Columbia (Col) for phenotypic analysis. This revealed normal plant phenotypes for most of the mutants; however, for the *NAD(P)-BINDING ROSSMANN-FOLD SUPER FAMILY PROTEIN* (*NBRSFP*; KO1, AT2G29300) and *SUCCINATE-SEMIALDEHYDE DEHYDROGENASE* (*SSADH1*, KO25, At1G79440) mutants the phenotype was dramatically altered (Fig. 2). After seed harvest seeds were tested for their seed performance phenotype.

### Altered seed dormancy for knock-out mutants in the dormancy and after-ripened up gene sets

**Fig. 3:**
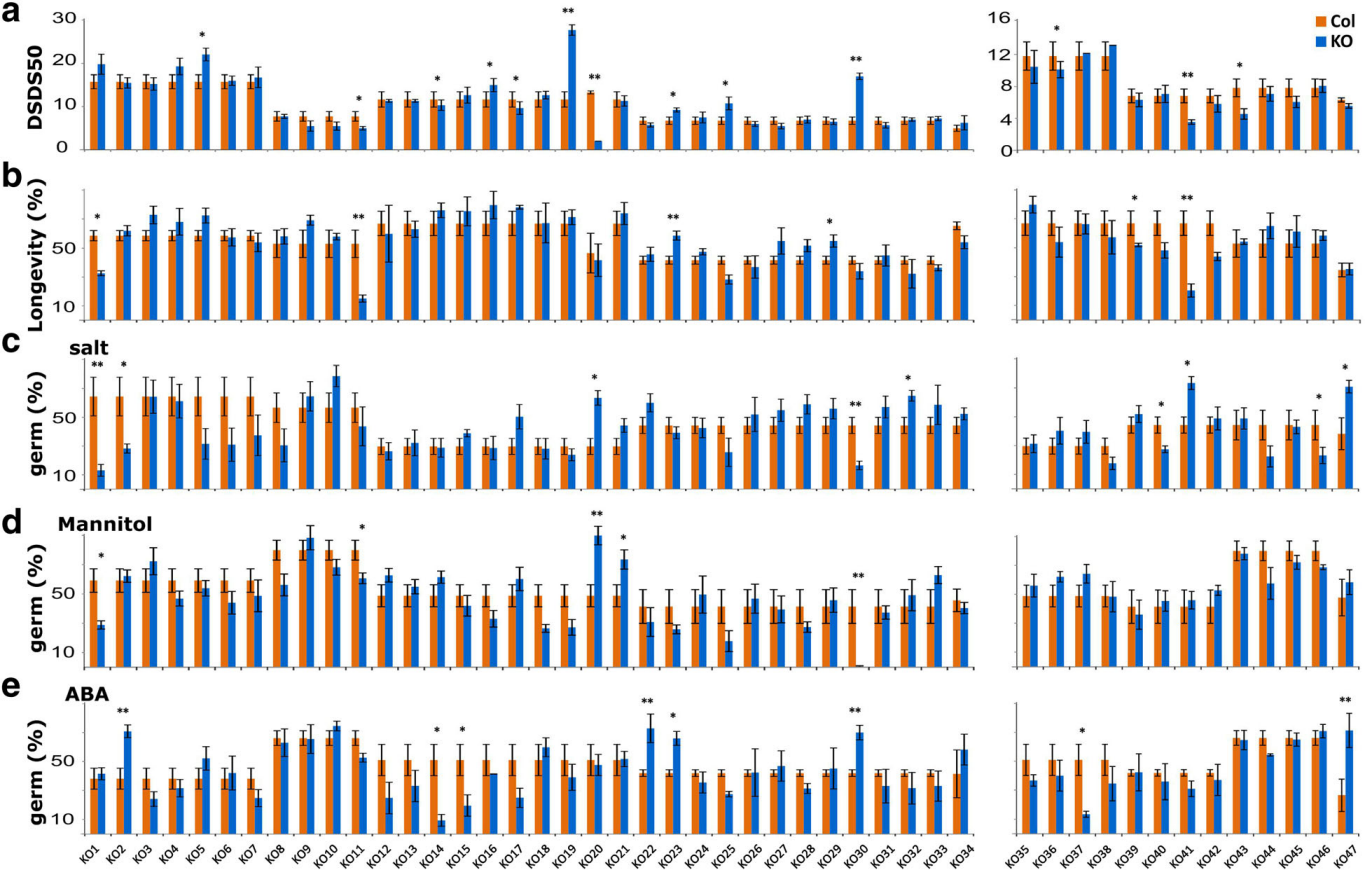
Germination behaviour of knock-out mutants (KO) in dormancy (left) and after –ripened (right) upregulated genes: (**a**) Average DSDS50 (Days of Seed Dry Storage until 50% germination) values. **b** germination after accelerated aging. **c** germination in salt 130 mM; d) in mannitol (−1 MPa) and e) in ABA(0.15 μM) solutions. Significant differences are indicated (*P < 0.05 and **P < 0.01). There are differences in Col-0 values between the different experiments, however, every knock-out line has been compared to the Col-0 that was grown in the same experiment

Initially only seed dormancy levels were examined by assessing the number of days of seed dry storage that were required to reach 50% of germination (Days of Seed Dry Storage to reach 50% of germination; DSDS50); Fig. 3a). Thereafter, the fully after-ripened seeds were tested for seed longevity (Fig. 3b) and germination in salt (Fig. 3c), mannitol (Fig. 3d) and ABA (Fig. 3e). Thirteen lines showed a dormancy level (DSDS50) that was significantly distinct from the wild type, of which seven were less dormant (KO11, 14, 16, 17, 20, 36, 41 and 43) and six were more dormant (KO5, 16, 19, 23, 25 and 30) than the wild type. Several of the mutants were specifically affected in their seed dormancy levels, so no other seed performance phenotypes were detected. Among these mutants were a transcription factor related gene which is known to respond to karrikin (KO5, At3G14880), *LECITHIN:CHOLESTEROL ACYLTRANSFERASE 3* (*LCAT3*; KO16, At3G03310) which is involved in lipid metabolism, a seed maturation protein (KO17; AT3G22490), *SSADH1*, *PIF6*, the antioxidant gene *PEROXIREDOXIN Q* (*PRXQ*; KO36, AT3G26060), and a *SEED STORAGE 2S ALBUMIN SUPER FAMILY MEMBER* (KO43, AT2G455180). *Pif6* displayed a more than two times higher DSDS50 (27.7 days) than the wild type (11.67). *PIF6* has been previously found to negatively regulate seed dormancy [47]. The other mutants were affected for at least one other seed performance trait, as well. Lines with mutations in *MALATE ENZYME 1* (*ME1*, KO11, At2G19900)*, ASG5* and an *unknown protein* (*PUF*, KO41, At4G31330) displayed both a dormancy and a seed longevity phenotype. Interestingly, *atnadp-me1* and *puf* had reduced dormancy and longevity and *puf* was also less sensitive to salt stress. *Asg5* showed increased dormancy and longevity. A KO in *ALLANTOATE AMIDOHYDROLASE* (*AAH*; KO30, AT4G20070) displayed a phenotype for all investigated seeds traits except for seed longevity. This gene encodes an enzyme that hydrolyses ureide allantoate to ureidoglycolate, CO2, and two molecules of ammonium. The *aah* mutant was more dormant, and more sensitive to salt and mannitol, but less sensitive to ABA. A KO in a *U-BOX SUPER FAMILY PROTEIN* (KO14, At2G27940) appeared slightly less dormant than wild type but was far more sensitive to ABA. A KO in the *UNCHARACTERISED PROTEIN FAMILY* gene (*UPF*; At4G19390, KO20) showed a very strong non-dormant phenotype, and was rather insensitive to mannitol and salt.

### Other seed performance phenotypes for D-up and AR-up genes

Among the selected mutants were also genotypes that were not affected in their seed dormancy levels but displayed altered phenotypes for other seed performance traits.

#### Mutants with altered seed longevity phenotype

The *nbrsfp* mutant (KO1, AT2G29300) is besides its reduced seed longevity, also more sensitive to germination in salt and mannitol. Lines mutated in *GIBBERELLIN-REGULATED GENE FAMILY* (*GASA3,* AT4G09600, KO29) and *UDP-GLUCOSYL TRANSFERASE 73B3* (*UGT73B3,* AT4G34131, KO39) showed a longevity phenotype. A role for these genes in seed longevity has not been reported previously.

#### Mutants with altered response to NaCl and/or osmotic stress

Lines mutated in mitochondrial *GLYOXALASE 2* (*GLX2*; KO2*,* AT2G31350), *DELTA VACUOLAR PROCESSING ENZYME* (*DELTA-VPE*, KO40, At3G20210) and *SERINE CARBOXYPEPTIDASE-LIKE 48* (*SCPL48*,KO46, AT3G45010) showed reduced germination in salt but tolerated low osmotic potentials caused by high concentrations of mannitol. Although more sensitive to salt, *glx2* was more resistant to ABA than wild type. The KO in *MYB TRANSCRIPTION FACTOR LHY-CCA1-LIKE1* (*LCL1*, KO21, AT5G02840) was more resistant to germination in mannitol compared to wild type. A similar trend was seen after germination in salt but this effect was not significant. Lines mutated in *CUPINS SUPER FAMILY PROTEIN* (KO32, AT4G36700) and *ALTERED XYLOGLUCAN 8* (AXY8; KO47, AT4G34260) showed a salt resistance phenotype but their germination in mannitol was similar to wild type. Besides lower sensitivity to salt, line *axy8* also showed reduced sensitivity to ABA.

#### Responses of dormancy related genes to ABA

Several lines that showed an altered response to ABA have already been mentioned above because they had at least also one other phenotype. However, there are three lines that showed a phenotype only for germination in the presence of ABA. Two lines, KOs in *HEAT SHOCK TRANSCRIPTION FACTOR A1E* (*HSFA1E*; KO15, AT3G02990) and *LOW AFFINITY PHOSPHATE TRANSPORTER* (*PHT2;1*, KO37,AT3G26570) were more sensitive, whereas the line mutated in *REGULATOR OF VPS4 ACTIVITY* in the MVB pathway protein (KO22, AT1G13340) was more resistant to ABA.

## Discussion

In our search for novel players in the regulation of *Arabidopsis* seed dormancy we employed a comparative transcriptomics approach for 24 h imbibed dormant and after-ripened *DOG*NILs and L*er* seeds. The same genotypes were earlier used to investigate the transcriptome of dormant dry seeds [6], which revealed that seed dormancy in the *DOG*NILs is mainly controlled by different additive genetic and molecular pathways. In dry seeds hardly any differences are found between dormant and after-ripened seeds, however as soon as seeds are being exposed to water, differences in the transcriptomes are evident. Based on these results we hypothesize that dormancy induction in the *DOG*NILs during seed maturation, for which dry seeds are the readout, is largely regulated by distinct molecular pathways, however dormancy maintenance during seed imbibition and the start of germination are likely very conserved processes. This conservation allowed us to identify a robust set of genes which are expressed at 24 h imbibed dormant and AR seeds. The genes identified depend a lot on the time-points chosen. From our earlier work we know that already at early imbibition (3 h after the start of imbibition) the first differences between dormant and after-ripened seeds can be identified [17]. However, we also know that most of changes in gene expression are related to seed rehydration itself and that those changes are similar between dormant and after-ripened seeds. To exclusively identify differences that are related to dormancy maintenance and germination we have chosen to investigate the transcriptome at 24 h after imbibition. This robustness of the identified genes was confirmed by comparison with previously published expression analysis that were performed with seeds of the Cvi accession at a range of physiological states [10, 17]. Of the dormancy and AR-up genes 63% and 32%, respectively, overlapped with genes that were identified by Cadman et al. [10] (Additional file 1: Table S1). Moreover, the D-up genes are also clearly higher expressed in dormant Cvi seeds as compared to AR Cvi seeds, as well as that the AR-up genes are on average higher expressed in AR seeds when compared to dormant seeds (Additional file 2: Figure S2). Some of the identified genes had previously been shown to play a role during germination and/or priming in several plant species. Among these are *GA3OX2* (AT1G80340) a key gene in the gibberellin biosynthesis pathway, *PIF6,* involved in the phytochrome signalling pathway and *ASG5* which is involved in protein and amino acid phosphorylation. The identification of these known dormancy mutants was the incentive to investigate the other dormancy and after-ripening specific genes. We took a reverse genetics approach by using T-DNA insertion lines for the differentially expressed genes and, indeed, we identified genes that had not been related to seed dormancy or germination before. Out of our target list of 66 genes, eight do not currently have any confirmed knock-out line available. This is consistent with a recent report that 12% of Arabidopsis genes do not have insertion lines available in previously generated collections [44]. The fact that a majority of the mutants showed near-wild type dormancy phenotypes, can be explained in several ways. The location of the T-DNA insertion may be decisive, e.g. whether in an intron, an exon or in untranscribed regions, such as promoters. Also, T-DNA–induced mutations do not always result in highly effective mutagenesis. Insertion in the protein-coding region of a gene generates a knockout in 86% of the cases and only 41% of the cases if the insertion is in front of the start codon [56]. Furthermore, gene redundancy may mask any phenotypic difference in plants in which the expression of only one homologue is disrupted [28]. In addition, in our experiments we used mutants with a Columbia-0 background that normally has low dormancy, which consequently does not allow the visualisation of small effects towards a decreasing dormancy level. Seed dormancy can be regulated by either inhibitory or promoting gene expression, considering the fact that in D-up genes we found both mutants that are less dormant (Fig. 3; KO11, 14 and 17) and more dormant (KO5, 16, 19, 23, 25 and 30). These examples demonstrate the inability to predict phenotypes based on expression pattern alone.

Many seed performance characteristics (i.e. seed desiccation tolerance, seed longevity and seed dormancy) are acquired during seed maturation. If genes affect seed maturation in general, it is likely that pleiotropic effects occur. In our study, some of the mutants showed a phenotype for more than one germination trait. The *aah* mutant for example displayed a phenotype for all investigated seed traits, except longevity. This enzyme degrades allantoate which is required to recycle purine-ring nitrogen in plants. The *aah* T-DNA mutant is unable to grow on allantoin as sole nitrogen source [57]. Furthermore, it is well known that conditions favouring nitrate accumulation in mother plant may lead to lower seed dormancy levels [1]. Since AAH is a key gene in the purine pathway [57], we speculate that defects in this gene block the pathway and, hence, availability of ammonium, resulting in increased primary dormancy and also affecting other seed performance traits of the mutant. *Atnadh-me1*, *asg5* and *ufp* mutants affected both dormancy and longevity and one additional trait. It is known that seed longevity can be a pleotropic effect of genes that regulate other traits, such as seed maturation [53], response to temperature [37], oxidative stress [14] and dormancy [7, 39]. Previous studies, using mutant analysis, have shown that the seed dormancy mutants *dog1* and *rdo4* also have a reduced seed longevity phenotype [7, 39].

Loss of dormancy is expressed as opening of the germination window (permissive range of environments) [19]. It is because of this that germination under stress (i.e. salt or osmotic stress) often correlates with initial seed dormancy levels. We revealed two cases for which reduced dormancy indeed coincided with reduced sensitivity to salt stress (*upf* (KO20) and *puf* (KO41)). For some mutants, *nbrsfp* (KO1), *upf* (KO20) and *aah* (KO30), germination patterns on both NaCl and mannitol correlated positively, probably because both treatments confer osmotic stress. Two of these mutants (*nbrsfp* and *aah*) displayed enhanced sensitivity to salinity and osmotic stress. The NAD(P)-binding Rossmann-fold superfamily protein has oxidoreductase activity, binding, catalytic activity and, based on TAIR annotation, it is located in the endomembrane system. *Upf* was the only mutant to be clearly more insensitive to both salt and osmotic stress, which indicates that this mutant is primarily osmotolerant. Furthermore, for some of the salt-tolerant mutants *RmlC-like cupins super family protein* (KO32), *unknown protein* KO41 and *axy8* (KO47) the germination rates on mannitol were similar to that of wild type. Likely, for the salt-tolerant lines, genes were mutated whose products are elements of stress signalling and inhibit germination under conditions of saline stress. The salt sensitive *glx2* mutant was more tolerant to the application of ABA. The glyoxalase pathway consists of the two enzymes GLX1 and GLX2 and has a vital role in chemical detoxification. In *Arabidopsis thaliana,* GLX2 is required during abiotic stress, as was concluded from the higher sensitive of *glx2-1*to salt stress and anoxia seeds compared to wild type seeds. Moreover, GLX2-1-OE seeds are more resistant to anoxic stress than wild type [18].

Interestingly, *axy8* (KO47) showed both a higher tolerance to ABA and to salt. *AXY8* encodes an α-fucosidase acting on hemicellulose xyloglucan (XyG) that occurs in the primary cell wall of all vascular plants. Due to its high levels in elongating tissues [11] and structural alterations during cell elongation [46], XyG has been proposed to be a major player in extension growth [15]. This was confirmed by the induction of genes involved in XyG metabolism during cell elongation and upon the addition of the growth hormone auxin [50]. Overall these findings emphasize the importance of cell wall remodelling in the germination process, especially in response to stress conditions.

In both the dormancy and after-ripened sets we identified many genes encoding enzymes. This result might be linked to the fact that we looked at 24-h imbibed seeds. At this stage most of the cells in the embryo are potentially metabolically active. This also activates hydrolytic and synthetic enzymes and growth hormones to mobilize nutrients and synthesize ingredients for growth. These include the genes encoding ABA- and GA-biosynthesis- and -deactivation enzymes that play critical roles in determining the ABA-GA balance in seeds, and hence, dormancy and germination [42, 58].

Among the identified genes are also nucleotide binding proteins and transcription factors, such as members of the heat stress transcription factor family (*HSFA1E*, KO15), members of the ERF transcription factor family, transcription factor-related protein (KO5), TRANSCRIPTION FACTOR HOMOLOGOUS TO ABI5 and *PIF6* (KO19). Interestingly all are found in the D-up state and for the ones that mutants were analysed they showed either more dormancy (*transcription factor-related protein* and *pif6*) or more sensitivity to ABA (*hsfa1a*).

## Conclusion

We identified seed dormancy and germination phenotypes for genes that had not been associated with seed dormancy before. We tested only one T-DNA allele per line which may not be a definitive prove that the insertional mutation causes the observed phenotype, as many as 50% of the lines may contain additional inserts at unknown loci [22, 44]. We identified germination related phenotypes for nearly 50% of the investigated genes, which is far higher compared to what can be expected from a random selection of genes. It is also far higher compared to the genotypes that we identified for genes identified in earlier transcriptome analyses. Nevertheless, it is possible that a second locus may cause the phenotype of interest, or may alter the phenotypic effect of a knockout mutation. This work therefor represented an inventory of genes that are likely involved in the control of seed dormancy or germination. However, in depth studies are required to reveal the molecular mechanism by which these genes affect these important seed traits.

## Additional files

Additional file 1: Figure S1. Microarray quality and reproducibility. All 28 ATH1 arrays used showed after hybridization similar patterns of intensity (A and B). Slide hybridization patterns were inspected manually without detecting artefacts. The RNAs used as templates for cRNA synthesis were shown to be intact based on Bio-analyzer 2001 analysis of both RNA template and biotinylated cRNA. In agreement with this were the hybridization patterns of control genes on the slide showing a near-identical pattern of hybridization (c). The uniformity of normalized unscaled SE (NUSE) and relative log expression (RLE) indicate high quality and uniformity of the hybridization data (d and e) (1). Raw intensity data were subjected to RMA normalization (2), which kept the uniformity of general levels between the different slides (f and g). Between replicate reproducibility of the experiment was high, exemplified by the high correlation between the data of two biological replicates (h). Array 1 and array 2 are hybridized with cRNA from different replicates of L*er* seeds. **Figure S2.** Spatial and temporal expression patterns of the selected dormancy and after-ripening up-regulated genes. Mean relative expression of (a) D-up and (b) AR-up genes across the Arabidopsis germination time course in the micropylar and chalazal endosperm (MCE) and radicle and hypocotyl (RAD) in dry, 1, 3, 7, 12, 16, 20, 25, 31 and 38 hours after imbibition. Data was taken from Seed EFP Browser (http://www.bioinformatics.nl/efp/cgi-bin/efpWeb.cgi). **Figure S3.** Log2 expression differences for the D-up and AR-up genes that are presented in Fig. 1c and d. (a) Heat map showing Log2 expression differences of the 245 NIL*DOG1* D-up genes (*P* < 0.0001) in NIL*DOG1* and the other genotypes. (b) Log2 expression differences of the 159 NIL*DOG1* AR-up genes (*P* < 0.0001) in NIL*DOG1* and the other genotypes. (XLS 81 kb)

Additional file 2: Table S1. T-DNA selection of the 46 D-up and 25 AR-up genes. Details like, T-DNA identification, genotype, primers used for genotyping, knock-out # in the analyses, where the T-DNA is inserted and whether the genes overlap with the study of Cadman et al. [10] are indicated. (DOCX 803 kb)

## Abbreviations

AAH: ALLANTOATE AMIDOHYDROLASE
ABA: Abscisic acid
ADS1: DELTA-9 DESATURASE1
AR: After-ripened
AR-up: After-ripening up-regulated genes
ASG5: ALTERED SEED GERMINATION 5
AXY8: ALTERED XYLOGLUCAN 8
Col: Columbia
DELTA-VPE: DELTA VACUOLAR PROCESSING ENZYME
*DOG*: *DELAY OF GERMINATION*
*DOG*NILs: *DELAY OF GERMINATION* near isogenic lines
DSDS50: Days of seed dry storage to reach 50% of germination
D-up: Dormancy up-regulated genes
FBA2: FRUCTOSE-BISPHOSPHATE ALDOLASE
GA3OX2: GIBBERELLIN 3-OXIDASE 2
GAs: Gibberellins
GASA3: GIBBERELLIN-REGULATED GENE FAMILY
GLX2: GLYOXALASE 2
HAS: Hours after sowing
HSFA1E: HEAT SHOCK TRANSCRIPTION FACTOR A1E
KO: Knock-out
LCAT3: LECITHIN:CHOLESTEROL ACYLTRANSFERASE 3
LCL1: MYB TRANSCRIPTION FACTOR LHY-CCA1-LIKE1
LEA: Late embryogenesis abundant
L*er*: Landsberg *erecta*
ME1: MALATE ENZYME
NBRSFP: NAD(P)-BINDING ROSSMANN-FOLD SUPER FAMILY PROTEIN
PHT2;1: LOW AFFINITY PHOSPHATE TRANSPORTER
PIF6: PHYTOCHROME-INTERACTING FACTOR 6
PRXQ: PEROXIREDOXIN Q
QTL: Quantitative trait loci
RIL: Recombinant inbred line
SCPL48: SERINE CARBOXYPEPTIDASE-LIKE 48
SSADH1: SUCCINATE-SEMIALDEHYDE DEHYDROGENASE
UGT73B3: UDP-GLUCOSYL TRANSFERASE 73B3
UPF: UNCHARACTERISED PROTEIN FAMILY
