# Supplementary material for: Differentially expressed genes during the imbibition of dormant and after-ripened seeds – a reverse genetics approach
Source: BMC Plant Biol. 2017 Sep 11;17:151. doi: 10.1186/s12870-017-1098-z (PMC5594490; doi:10.1186/s12870-017-1098-z)
Supplement: Supplementary file 2 — T-DNA selection of the 46 D-up and 25 AR-up genes. Details like, T-DNA identification, genotype, primers used for genotyping, knock-out # in the analyses, where the T-DNA is inserted and whether the genes overlap with the study of Cadman et al. [10] are indicated. (DOCX 803 kb) [file 12870_2017_1098_MOESM2_ESM.docx]

**
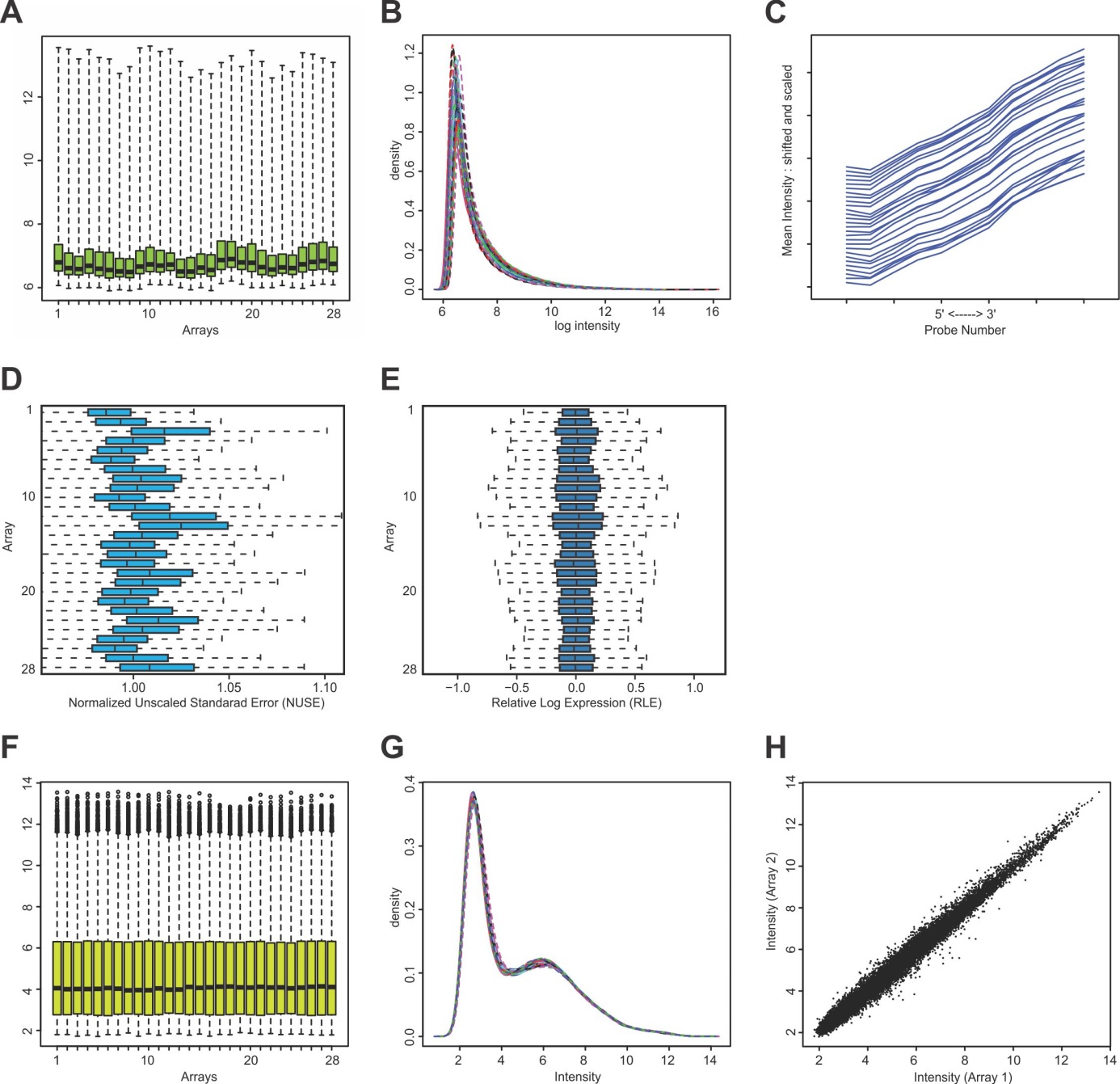
Additional file 2: Figure S1**. Microarray quality and reproducibility. All 28 ATH1 arrays used showed after hybridization similar patterns of intensity (A and B). Slide hybridization patterns were inspected manually without detecting artefacts. The RNAs used as templates for cRNA synthesis were shown to be intact based on Bio-analyzer 2001 analysis of both RNA template and biotinylated cRNA. In agreement with this were the hybridization patterns of control genes on the slide showing a near-identical pattern of hybridization (C). The uniformity of normalized unscaled SE (NUSE) and relative log expression (RLE) indicate high quality and uniformity of the hybridization data (D and E) (1). Raw intensity data were subjected to RMA normalization (2), which kept the uniformity of general levels between the different slides (F and G). Between replicate reproducibility of the experiment was high, exemplified by the high correlation between the data of two biological replicates (H). Array 1 and array 2 are hybridized with cRNA from different replicates of L*er* seeds.

**
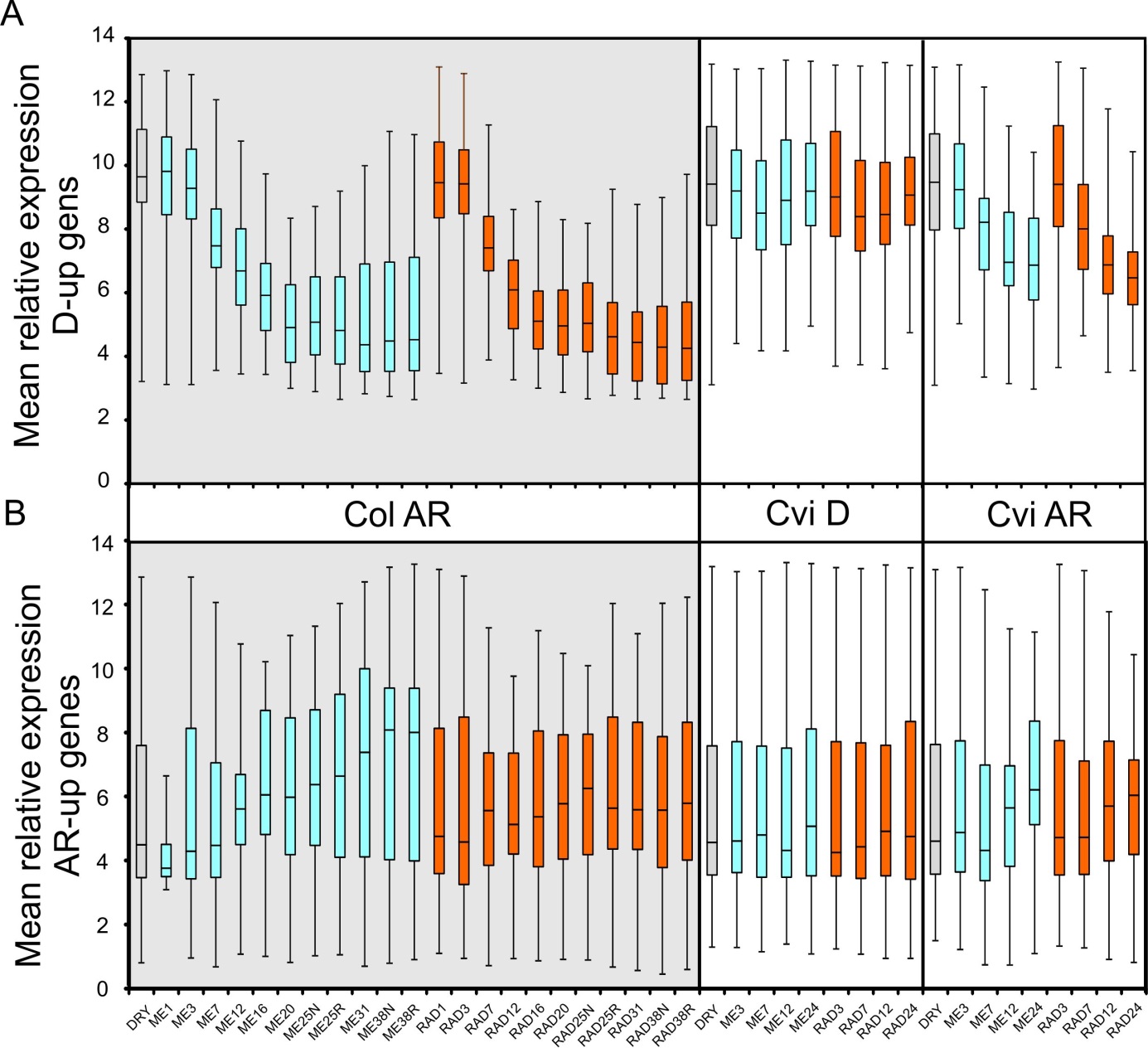
**

**Additional file 2: Figure S2**. Spatial and temporal expression patterns of the selected dormancy and after-ripening up-regulated genes. Boxplots of mean relative expression of A) D-up and B) AR-up genes in after-ripened (AR) Columbia (Col; grey box)) and dormant and AR Cape Verde Island (Cvi; white box) seeds across the germination time course. Expression within the micropylar endosperm (ME, blue bars) and radicle (RAD, orange bars) are shown at different time-points of seed imbibition (i.e. RAD7, radicle at 7 hours of imbibition, N is non-ruptured, R is ruptured). Data was taken from Seed EFP Browser (http://www.bioinformatics.nl/efp/cgi-bin/efpWeb.cgi).


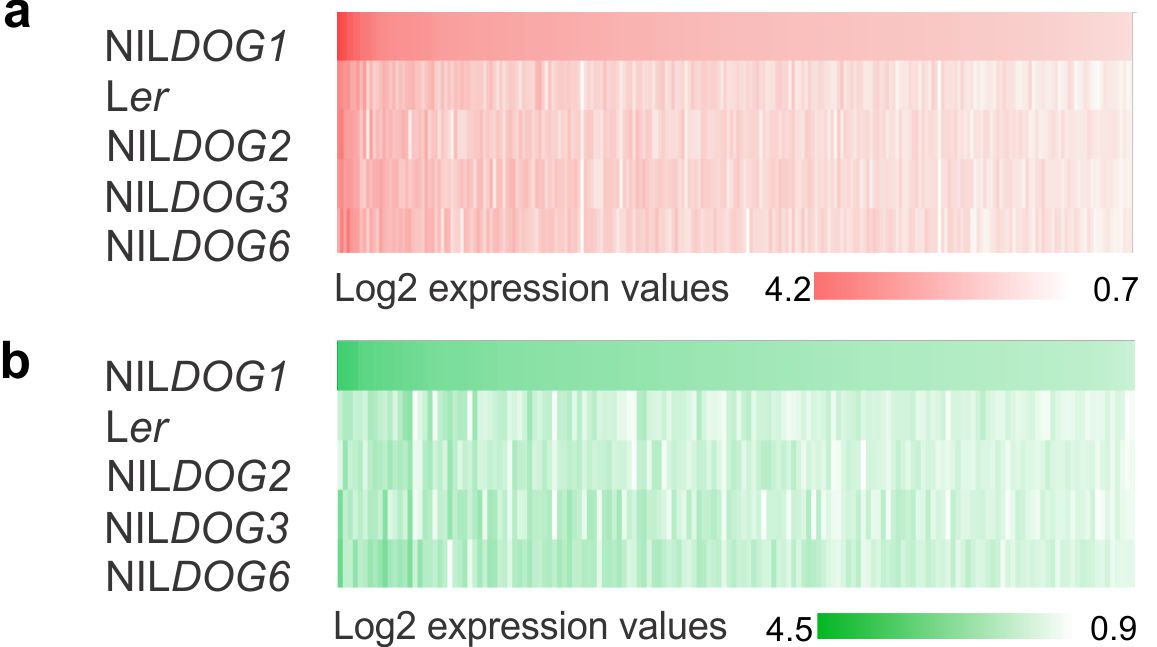


**Additional file 2: Figure S3**. Log2 expression differences for the D-up and AR-up genes that are presented in Figure 1c and d. (a) Heat map showing Log2 expression differences of the 245 NIL*DOG1* D-up genes (P < 0.0001) in NIL*DOG1* and the other genotypes. (b) Log2 expression differences of the 159 NIL*DOG1* AR-up genes (P < 0.0001) in NIL*DOG1* and the other genotypes.
